# Species Differences in Microsomal Metabolism of Xanthine-Derived A_1_ Adenosine Receptor Ligands

**DOI:** 10.3390/ph14030277

**Published:** 2021-03-18

**Authors:** Daniela Schneider, Dirk Bier, Marcus Holschbach, Andreas Bauer, Bernd Neumaier

**Affiliations:** 1 Institute of Neuroscience and Medicine—Molecular Organization of the Brain (INM-2), Forschungszentrum Jülich GmbH, 52428 Jülich, Germany; an.bauer@fz-juelich.de; 2 Institute of Neuroscience and Medicine—Nuclear Chemistry (INM-5), Forschungszentrum Jülich GmbH, 52428 Jülich, Germany; d.bier@fz-juelich.de (D.B.); m.holschbach@fz-juelich.de (M.H.); b.neumaier@fz-juelich.de (B.N.); 3 Neurological Department, Medical Faculty, Heinrich-Heine-University, Universitätsstraße 1, 40225 Düsseldorf, Germany

**Keywords:** A_1_ adenosine receptor, liver microsomes, metabolism, radioligand, species differences, preclinical evaluation

## Abstract

Tracer development for positron emission tomography (PET) requires thorough evaluation of pharmacokinetics, metabolism, and dosimetry of candidate radioligands in preclinical animal studies. Since variations in pharmacokinetics and metabolism of a compound occur in different species, careful selection of a suitable model species is mandatory to obtain valid data. This study focuses on species differences in the in vitro metabolism of three xanthine-derived ligands for the A_1_ adenosine receptor (A_1_AR), which, in their ^18^F-labeled form, can be used to image A_1_AR via PET. In vitro intrinsic clearance and metabolite profiles of 8-cyclopentyl-3-(3-fluoropropyl)-1-propylxanthine (CPFPX), an established A_1_AR-ligand, and two novel analogs, 8-cyclobutyl-3-(3-fluoropropyl)-1-propylxanthine (CBX) and 3-(3-fluoropropyl)-8-(1-methylcyclobutyl)-1-propylxanthine (MCBX), were determined in liver microsomes from humans and preclinical animal species. Molecular mechanisms leading to significant differences between human and animal metabolite profiles were also examined. The results revealed significant species differences regarding qualitative and quantitative aspects of microsomal metabolism. None of the tested animal species fully matched human microsomal metabolism of the three A_1_AR ligands. In conclusion, preclinical evaluation of xanthine-derived A_1_AR ligands should employ at least two animal species, preferably rodent and dog, to predict in vivo behavior in humans. Surprisingly, rhesus macaques appear unsuitable due to large differences in metabolic activity towards the test compounds.

## 1. Introduction

The development of novel radioligands for imaging molecular targets via positron emission tomography (PET) is a time-consuming and costly endeavor. In particular, assessment of imaging characteristics and safety of a candidate compound requires extensive preclinical investigations prior to initial clinical trials. Pharmacokinetics and metabolism are important determinants of the in vivo properties of a novel imaging agent [1]. Radioligand metabolism can lead to radioactive metabolites that impede reliable quantification of the molecular target. Rapid blood clearance of the radioligand may limit target exposure, but on the other hand can enhance the signal-to-background ratio by reducing the amount of radioactivity present in the vascular system [2]. Metabolism also plays a critical role with regard to the safety of radiopharmaceuticals, as it strongly influences the radiation doses received by individual organs and tissues of the test subjects or patients [3]. Since most PET radioligands are small lipophilic molecules, metabolism is crucial for the excretion of these compounds from the body and largely determines their biological half-lives. On these grounds, metabolism studies are indispensable prerequisites for the selection and optimization of radioligand candidates. As with conventional drugs, radioligand metabolism is typically evaluated using in vitro systems and preclinical animal models. Since inter-species differences in the metabolism of a compound can be significant, careful selection of the appropriate species for preclinical studies is essential to obtain data that can be extrapolated to human metabolism.

The xanthine-derived radioligand 3-(3-[^18^F]fluoropropyl)-1-propylxanthine ([^18^F]CPFPX, structural formula given in Table 1) is an established radiotracer for PET imaging of A_1_ adenosine receptors (A_1_AR) [4,5]. [^18^F]CPFPX is rapidly metabolized in humans and rodents, as reflected in a fast decline of plasma concentrations and formation of numerous radiometabolites [6]. One of these metabolites, a difunctionalized oxo-desaturation product (“enone metabolite”; structural formula given in Table 1), has been identified as problematic for PET imaging due to its slow excretion from the body. Although this metabolite does not penetrate the blood–brain barrier, its accumulation in the vascular compartment leads to increased background noise and radiation exposure. For these reasons, continuous efforts have been made to develop [^18^F]CPFPX analogs with higher metabolic stability producing no radiometabolites with long biological half-lives [7].

Early [^18^F]CPFPX in vitro studies using human and rodent hepatic microsomes revealed that human biotransformation of the radioligand differs from rodent biotransformation with regard to the aforementioned enone radiometabolite [6]. More specifically, there was extensive formation of the metabolite in human microsomes, whereas almost no significant amounts could be detected in microsomes from rats and mice. However, plasma analyses of rats and mice that received [^18^F]CPFPX injections clearly showed that the enone metabolite is generated in vivo, raising questions regarding the validity of the in vitro model and its suitability for evaluation of this compound class.

The objectives of the present study were two-fold: first, to elucidate the mechanisms underlying the distinct in vitro and in vivo metabolite profiles of CPFPX observed in rodents but not humans; second, to investigate species differences in the metabolism of CPFPX and two novel cyclobutyl analogs, namely 8-cyclobutyl-3-(3-fluoropropyl)-1-propylxanthine (CBX, see Table 1) and 3-(3-fluoropropyl)-8-(1-methylcyclobutyl)-1-propylxanthine (MCBX, see Table 1), in hepatic microsomes from humans and commonly used preclinical animal species with the aim to identify suitable animal models for evaluation of xanthine-derived A_1_AR radioligands.

## 2. Results

### 2.1. In Vitro Intrinsic Clearance

**Figure 1:**
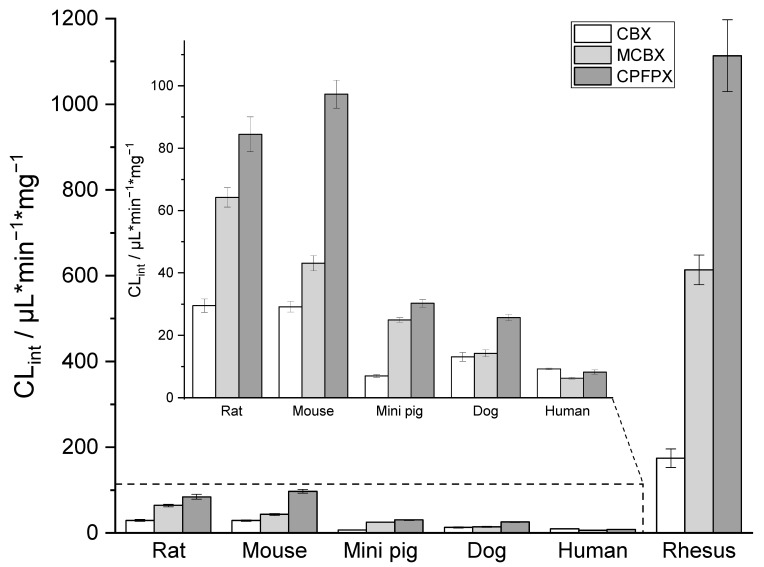
Comparison of CBX, MCBX, and CPFPX intrinsic clearance in liver microsomes from various species. Data represent the mean ± SD of four independent experiments.

In vitro intrinsic clearance (CL_int_) of CBX, MCBX, and CPFPX was determined using human, rat, mouse, dog, mini pig, and rhesus monkey liver microsomes (Figure 1). Mean CL_int_ values for the compounds varied widely across species. Highest metabolic activity was observed in rhesus microsomes, with up to 134-fold higher CL_int_ values (CPFPX) than in human microscomes. The metabolic activities of mini pig and dog microsomes were roughly comparable to each other, as were those of rat and mouse microsomes. The rank order of CL_int_ was CPFPX > MCBX > CBX in microsomes from rat, mouse, mini pig, and rhesus; CPFPX > MCBX ≈ CBX in dog microsomes; and CBX > CPFPX > MCBX in human microsomes.

### 2.2. Metabolite Profiles

**Figure 2:**
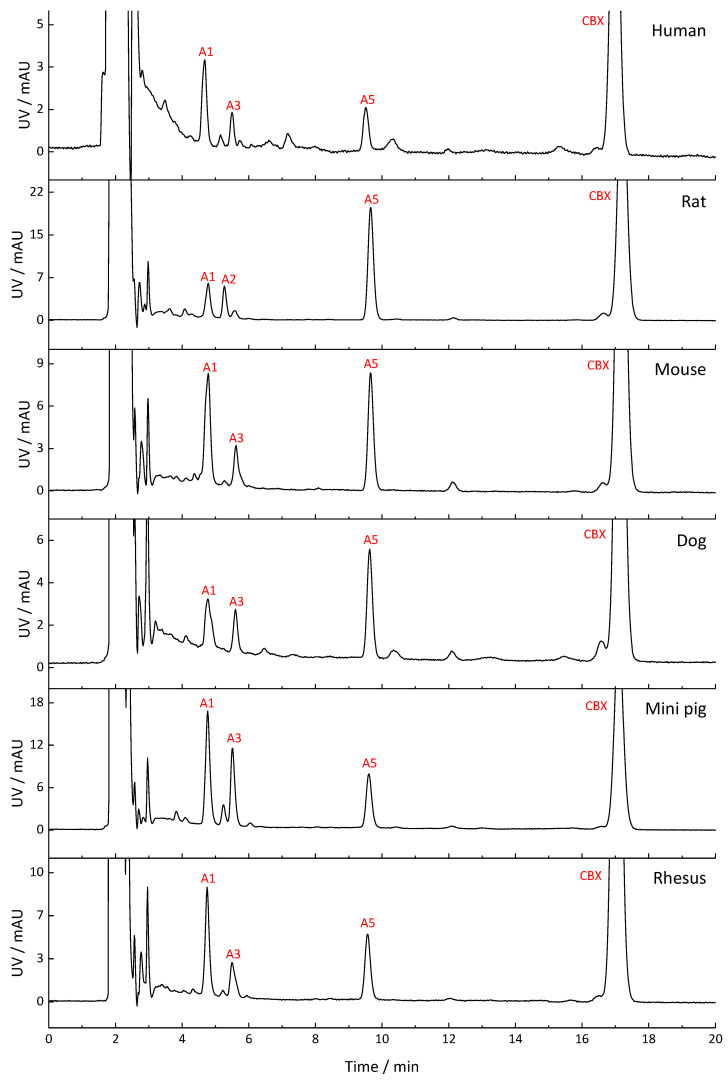
Metabolite profiles of CBX generated in liver microsomes from humans and different animal species. Detection wavelength was 275 nm. In the chromatograms, only metabolite peaks accounting for at least 10% of the total metabolite peak area are labeled. A comprehensive list of the detected metabolites can be found in Table 2.

**Figure 3:**
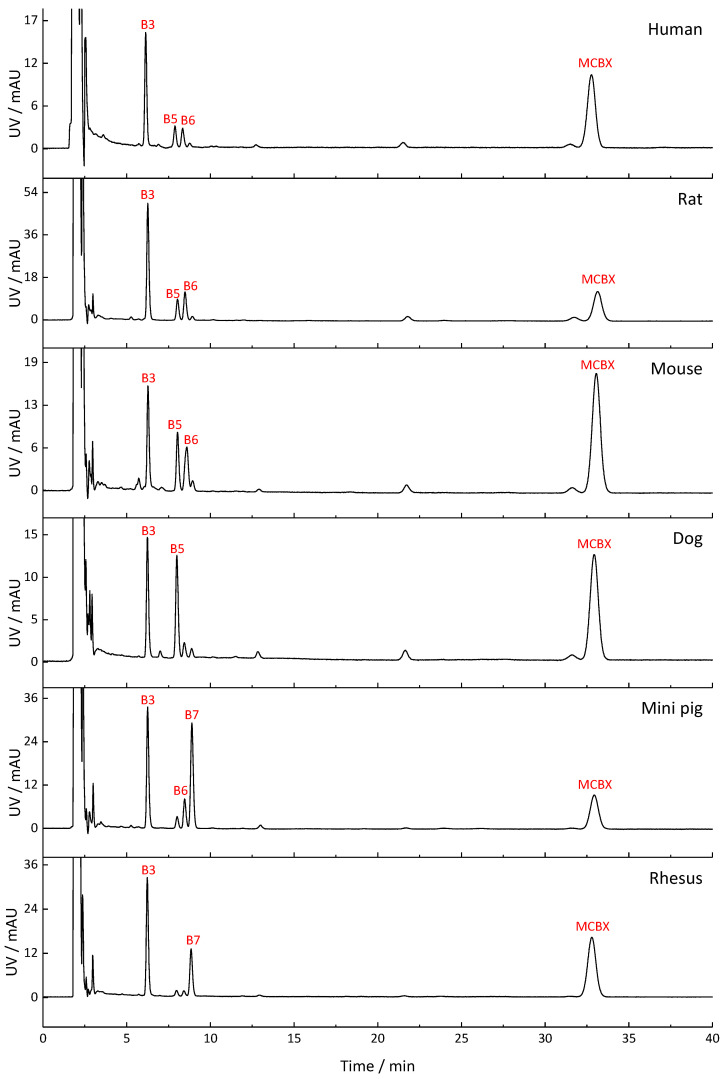
Metabolite profiles of MCBX generated in liver microsomes from humans and different animal species. Detection wavelength was 275 nm. In the chromatograms, only metabolite peaks accounting for at least 10% of the total metabolite peak area are labeled. A comprehensive list of the detected metabolites can be found in Table 3.

**Figure 4:**
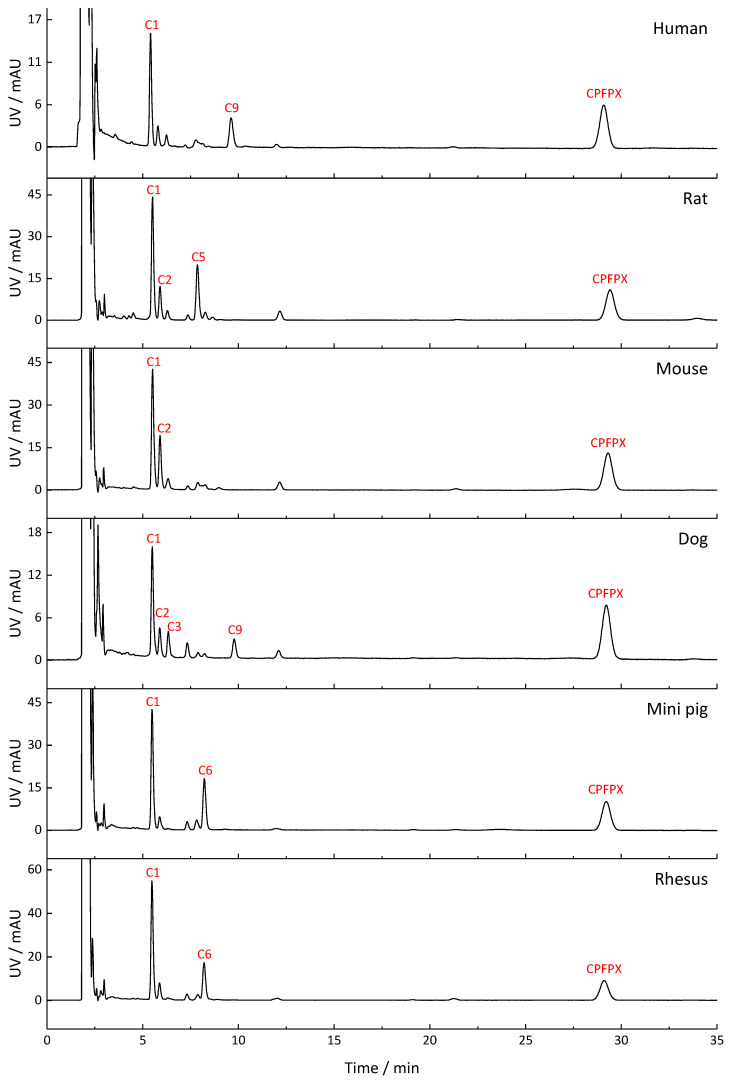
Metabolite profiles of CPFPX generated in liver microsomes from humans and different animal species. Detection wavelength was 275 nm. In the chromatograms, only metabolite peaks accounting for at least 10% of the total metabolite peak area are labeled. A comprehensive list of the detected metabolites can be found in Table 4.

**Figure 5:**
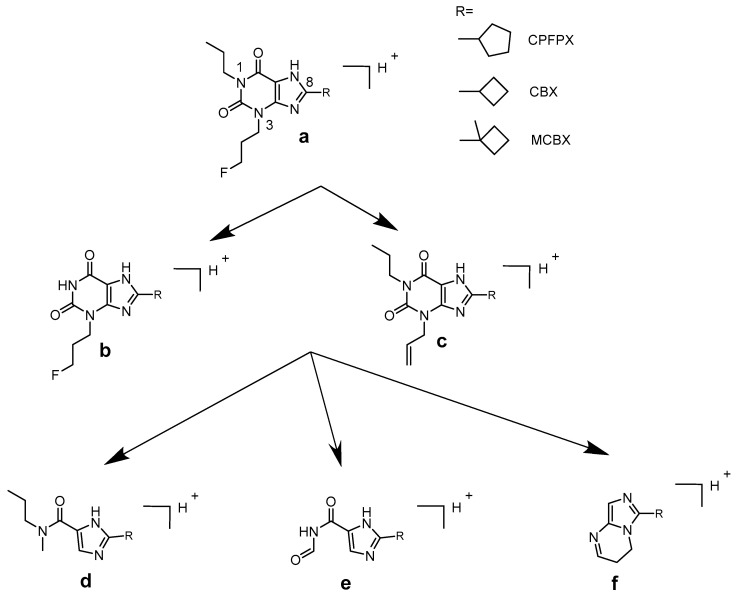
Base fragments (**a**–**f**) for interpretation of the in-source fragmentation pathways of the C8-substituted xanthine compounds.

The metabolite profiles of CBX, MCBX, and CPFPX generated by human, rat, mouse, dog, mini pig, and rhesus monkey liver microsomes are compared in Figure 2, Figure 3 and Figure 4. Metabolites were distinguished from matrix components by comparison with blank samples and by mass spectrometric analysis. Whenever possible, peak identities (type and site of functionalization) were derived from the mass spectra. For interpretation of the in-source fragmentation patterns observed at a cone voltage of 185 V, experiences gained during previous LCMS studies were taken into account [6,8]. Plausible fragmentation routes are shown in Figure 5. The assigned metabolites are listed in Table 2, Table 3 and Table 4, together with their functionalization. Monohydroxylation represented the main route of biotransformation for all three compounds. Functionalization predominantly occurred at the cyclic C8-moiety, as revealed by the in-source fragmentation patterns.

Metabolism of CBX produced up to nine metabolites in the microsomes of the test species, of which the hydroxylated compounds A1 and A5 were dominant in all species. Two metabolites which coeluted at R_t_ = 5.6 min could be distinguished via mass spectrometry. Metabolite profiles of rhesus monkey and mini pig exhibited the highest degree of similarity to the human metabolite profile. Microsomal metabolism of MCBX generated up to 10 metabolites. Metabolite B3, a compound resulting from monohydroxylation of the cyclobutyl ring, represented the main metabolite in all species. Metabolites B5–B7, which were also identified as hydroxylated metabolites, occurred in the profiles of all species but in differing proportions. Rodent metabolite profiles of MCBX most closely resembled their human counterpart. In vitro metabolism of CPFPX in microsomes of humans and rodents has already been studied by Bier et al. [6]. To complement this previous work, CPFPX metabolism in microsomes of three non-rodent species was investigated in the current study in order to identify the most suitable animal model for human metabolism of xanthine-derived A_1_AR ligands. Species-specific microsomal metabolism of CPFPX resulted in up to 12 metabolites. Metabolite C9, which was identified as the enone metabolite **4** ([M+H]^+^ = 335), was generated exclusively in human and dog microsomes.

**Figure 6:**
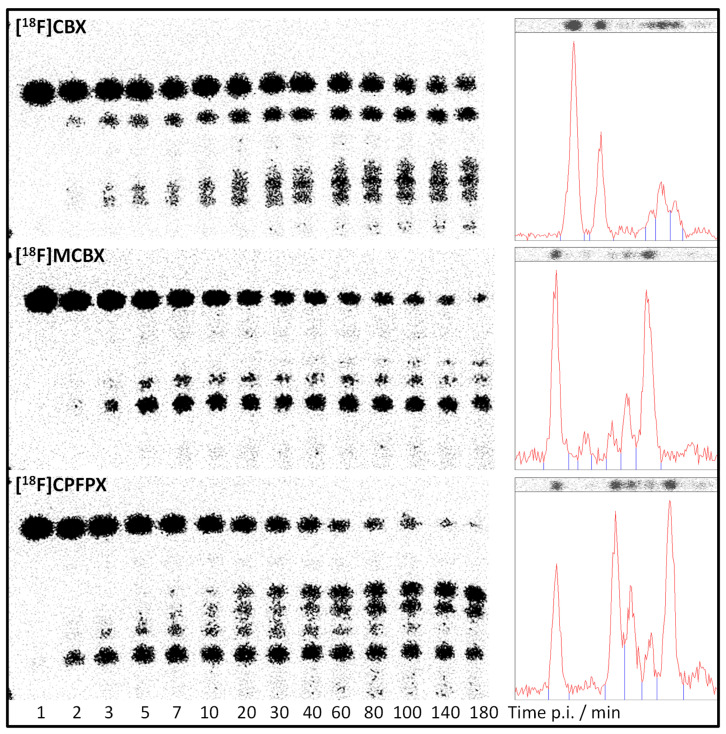
In vivo metabolite profiles of [^18^F]CBX, [^18^F]MCBX, and [^18^F]CPFPX obtained from rat plasma. Left panels: radio-TLC images (SIL G-25 plates); right panels: corresponding densitometer scans at 60 min post-injection.

As part of a previous study aimed at comparing the PET imaging characteristics of [^18^F]CBX, [^18^F]MCBX and [^18^F]CPFPX, in vivo metabolite profiles were generated from rat plasma [7]. Representative radio-thin layer chromatograms (radio-TLCs) are shown in Figure 6. Although metabolite identification was beyond the scope of the cited study, visual comparison of the radio-TLCs and the HPLC-UV chromatograms shown in Figure 2, Figure 3 and Figure 4 reveals a high degree of similarity with regard to peak number (peaks of considerable size), peak areas, and elution order.

### 2.3. Enone Metabolite Formation in Liver Microsomes

In vitro formation of enone metabolite **4** was investigated by incubation of human and rat liver microsomes with four primary metabolites (**5**–**8**, see Table 1) present in the human microsomal metabolite profile, which could potentially serve as precursors for enone formation.

**Figure 7:**
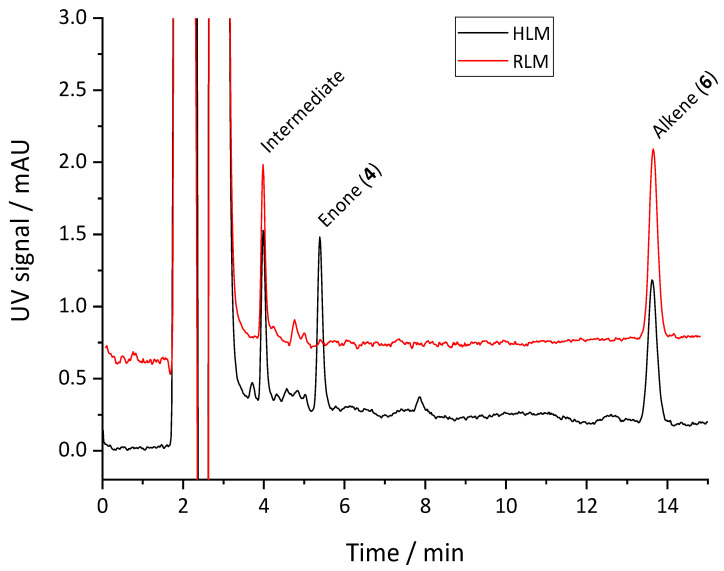
Metabolism of **6** in human (black) and rat (red) liver microsomes. Formation of **4** occurs exclusively in human liver microsomes. Chromatograms were generated at 275 nm and smoothed using a moving average algorithm (filter width 2).

Incubation of **5**–**8** with human liver microsomes (HLM) revealed exclusive formation of **4** from precursor **6**. During this biotransformation process, a stable intermediate was generated, which could be separated by HPLC (Figure 7). Mass spectrometry showed that this molecule contains a cyclopentenol ring instead of the cyclopentenone ring of **4**, thus being 3-(3-fluoropropyl)-8-(3-hydroxycyclopent-1-en-1-yl)-1-propylxanthine (**9**).

**Figure 8:**
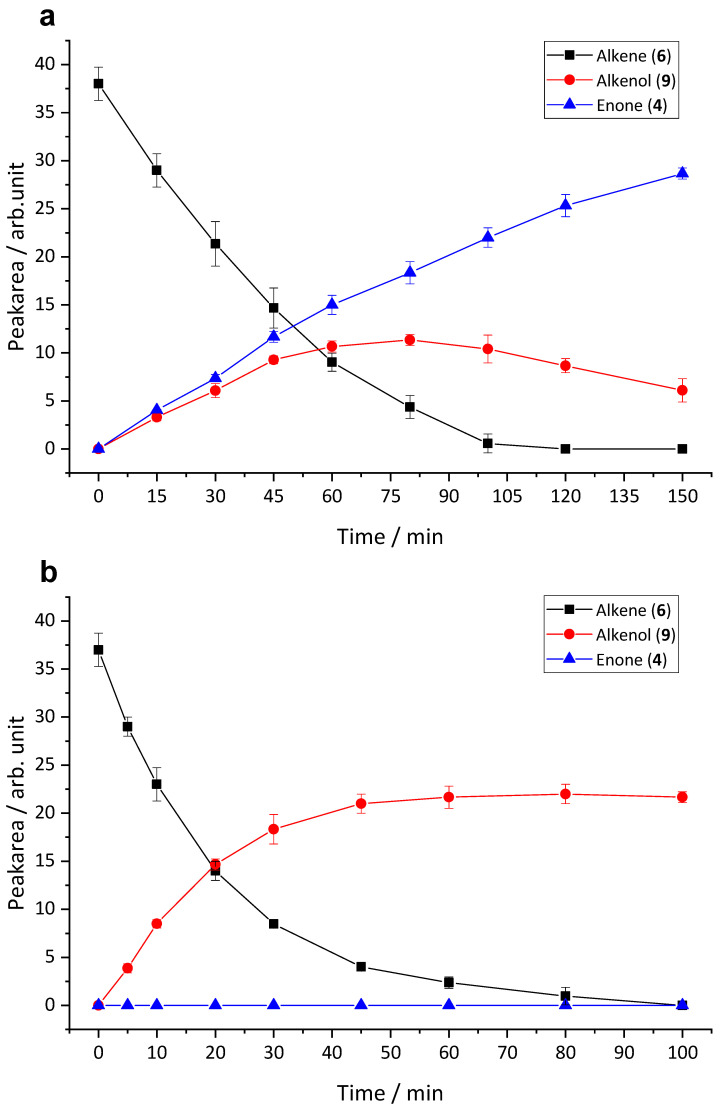
Time course of metabolism of **6** in microsomes from humans (**a**) and rats (**b**). In both species, metabolism of **6** (black curve) generates an intermediate alkenol **9** (red curve). In human but not in rat liver microsomes, **9** is further metabolized to **4** (blue curve). Data points represent the mean ± SD of three independent experiments.

**Figure 9:**
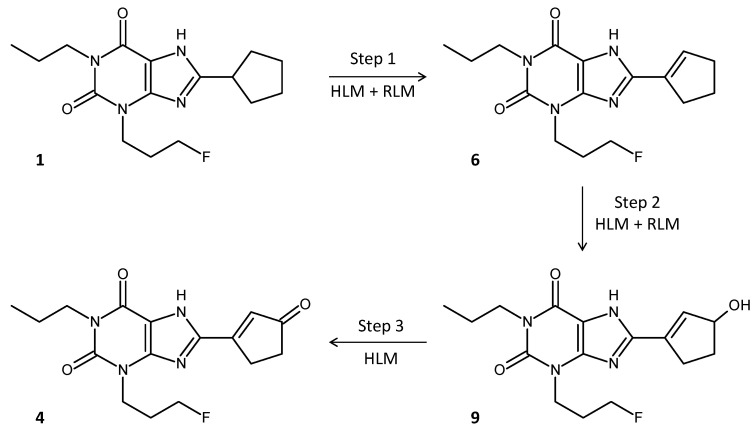
Proposed biotransformation pathway of CPFPX (**1**) in human liver microsomes. A three-step oxidation sequence transforms the parent compound into the enone metabolite (**4**). In rat liver microsomes, only reaction steps 1 and 2 take place, but not the final oxidation step 3, which converts the hydroxy intermediate (**9**) into the enone.

The time course of formation of **4** and **9** in HLM is shown in Figure 8a. It is evident from the curves that the formation of **4** still proceeded after complete depletion of **6**, whereas the concentration of **9** started to decline after a certain incubation time. These observations indicate that the biotransformation proceeds from **6** via **9** to **4** (Figure 9). For comparison, the time course of metabolism of **6** in rat liver microsomes (RLM) is given in Figure 8b. As with HLM, **6** was oxidized to produce **9**, however, the subsequent oxidation step resulting in ketone formation obviously did not occur. Consequently, the concentration of **9** increased during incubation until **6** was completely depleted but then remained constant.

## 3. Discussion

Generally speaking, species differences in microsomal metabolism can be related to several factors, which comprise variations in the levels of total microsomal P450 or individual P450 isoforms as well as differences in the mechanistic aspects of catalytic enzyme function (substrate specificity, catalytic activity, main reaction pathways).

Species differences in the rate of substrate metabolism resulting from varying levels of total or individual P450 enzymes in microsomal preparations can frequently be compensated by adjustment of the protein concentration used in the assay or by introduction of scaling factors. Variations in the functional characteristics of enzymes may, however, lead to fundamental differences in metabolism, rendering a particular animal species unsuitable for prediction of human xenobiotic metabolism in preclinical studies.

In this study, hepatic microsomes were chosen as analytical model for comparison of species-specific enzyme function. Although microsomes are considered a less physiologically relevant model than hepatocytes due to the lack of cellular organization, they are still a valuable tool for clearance determination of compounds that are metabolized primarily by phase I enzymes and that do not act as transporter substrates. Results from previous studies showed that xanthine-derived A_1_AR ligands are metabolized primarily by hepatic P450 enzymes [9] and that scaled microsomal clearance data are in good agreement with measured in vivo clearance [10]. Against this background, hepatic microsomes were preferred over hepatocytes for investigating species differences in A_1_AR ligand metabolism. In addition, for rapidly metabolized substrates such as CPFPX, measurements conducted with hepatocytes could potentially provide biased results due to the capacity/rate limitation of the hepatocyte system [11,12].

Regarding the total P450 content of the microsomes used in the present study, manufacturer specifications were only available for human preparations. For the non-human animal species, literature data on total microsomal P450 concentrations were used as reference values for the further discussion of the results (see Table 5).

Phase I metabolism of the xanthine-based compound CPFPX has been shown to be mainly governed by P450 1A2 [9], so the following discussion will focus on species differences in expression levels and functional characteristics of this specific P450 isoform. While data on hepatic P450 1A2 levels are currently not available for all species used in the present study, it has been shown that P450 1A2 accounts for about 13% of the total P450 content in humans [18], for about 2% in rats [19], and for about 4% in dogs [20]. Basal hepatic levels of P450 1A2 in macaques are generally reported to be low or even undetectable [21,22,23,24,25], except for the data published by Shimada et al. which showed similar P450 1A2 levels in hepatic microsomes of cynomolgus macaques and humans [13]. Evidently, the pronounced interspecies differences in microsomal metabolism of the tested A_1_AR ligands cannot be readily explained by varying levels of total and isoform-specific P450. Microsomal P450 levels reported in Table 5 are about 2–3 times higher in rodents and about 3 times higher in rhesus and cynomolgus monkeys than in humans. By contrast, CL_int_ of CPFPX in rodent and rhesus monkey microsomes exceeded human values by a factor of about 10 and 134, respectively. This is all the more remarkable in view of the questionable constitutive expression of P450 1A2 in rhesus monkey liver. It is conceivable that P450 isoforms other than 1A2 are mainly responsible for biotransformation of CPFPX and its analogs in rhesus microsomes, casting doubt on the usefulness of macaques as preclinical species for pharmacokinetic evaluation of xanthine-derived A_1_AR ligands.

Published data on species differences in the microsomal metabolism of various P450 1A2 marker substrates reveal large differences in enzymatic activity and substrate specificity of the homologous 1A2 enzymes [13,26,27,28,29,30,31,32]. Interestingly, the rate of caffeine metabolism, which was studied by Berthou et al., was considerably higher in hepatic microsomes from humans than in those from rats or monkeys [26], which is in contrast to the results obtained in the present study. However, these discrepancies might be a result of the different qualities of human microsomal preparations used for determination of metabolic stability. In this study, microsomal assays were conducted with pooled microsomes (50 donors) to minimize individual variations in enzyme levels and activities. By contrast, the microsomal material used by Berthou et al. was obtained from liver tissue of a single donor and showed a particularly high P450 level [33], which might have introduced bias into the assessment of microsomal enzyme activity.

In summary, rates of CBX, MCBX, and CPFPX metabolism in hepatic microsomes are highly species-dependent. Simple scaling approaches are not sufficient to overcome these issues. The rank order of metabolic stability in human microsomes did not correspond to any of the animal species, which raises considerable doubts regarding the relevance of animal microsomes as a model for human pharmacokinetics of the test compounds. However, it should be noted that the specific conditions encountered during sampling and preparation of human liver tissue could potentially result in biased metabolism data. First, biopsy specimens might be altered pathologically. Second, prolonged post-mortem times (resulting from, e.g., regulatory requirements) encountered during preparation of human tissue can lead to rapid loss of enzyme activity because of autolysis [34,35,36,37,38]. To date, there still is little profound knowledge regarding the implications of post-mortem enzyme degradation on microsomal stability data.

Metabolite profiles of the test compounds varied considerably among species. Biotransformation of both CBX and MCBX resulted in similar metabolites in microsomes of all test species, but in highly variable ratios. Regarding CPFPX metabolism, metabolite **4** (enone) was generated exclusively in human and dog microsomes. Enone formation is a dominant pathway in human CPFPX metabolism [6,9]; therefore, prevention of this reaction sequence might be a promising strategy to develop CPFPX analogs with higher metabolic stability. In rodents, **4** is only generated in vivo, but not in microsomes [6]. It could be demonstrated that in liver microsomes of rats, as opposed to human microsomes, the final oxidation step of the biotransformation pathway does not take place (at least not to a significant extent). It is likely that the presence or absence of distinct metabolic pathways results from species differences in the functional properties of P450 1A2, which can probably be attributed to variations in active site structure. In vivo formation of **4** in living rats may reflect the catalytic action of other hepatic or extrahepatic enzyme systems (e.g., extrahepatic P450 isoenzymes or alcohol oxidoreductases) to which intermediate metabolites are subjected via systemic circulation. To elucidate the exact mechanism of in vivo formation of **4** in rodents as well as the particular enzyme systems involved in this pathway, further studies including the investigation of CPFPX metabolism by hepatocytes, intestinal microsomes, and individual isoenzymes are planned.

## 4. Materials and Methods

### 4.1. Compounds

All compounds listed in Table 1 were synthesized and characterized in our laboratories according to the procedures described in [4,10,39,40,41].

### 4.2. Reagents and Solvents

Reduced β-nicotinamide adenine dinucleotide 2′-phosphate (NADPH) was supplied by Roche Diagnostics (Mannheim, Germany). Dimethyl sulfoxide (DMSO), 4-(2-hydroxyethyl)-1-piperazineethanesulfonic acid (HEPES), magnesium chloride (MgCl_2_), sodium hydroxide (NaOH), and acetic acid (AcOH) were obtained from Sigma-Aldrich (Steinheim, Germany). Reagent-grade acetonitrile (MeCN) and methanol (MeOH) were purchased from Merck (Darmstadt, Germany). Aqua ad iniectabilia (water for injection) from B. Braun Melsungen (Melsungen, Germany) was used for preparation of buffers and eluents.

### 4.3. In Vitro Studies

#### 4.3.1. Determination of In Vitro Intrinsic Clearance

Liver microsomes from Sprague Dawley rats (RLM), CD-1 mice (MLM), beagle dogs (DLM), Göttinger mini pigs (MPLM), rhesus monkeys (RMLM), and humans (HLM, specified total P450 content: 0.286 nmol/mg protein) were obtained from Thermo Fisher Scientific/Life Technologies (Darmstadt, Germany). Optimization of incubation conditions (microsomal protein concentration, substrate solvent, incubation buffer) has been carried out in a previous study [41]. For assessment of intrinsic clearance (CL_int_), substrate (8 µM CBX, MCBX or CPFPX, stock solutions in DMSO) and microsomes (0.4 mg/mL RLM, MLM, DLM and MPLM, 2.0 mg/mL HLM and 0.04 mg/mL RMLM) were preincubated for 5 min at 37 °C in HEPES buffer (100 mM, pH 7.4) containing MgCl_2_ (3.3 mM). Enzymatic reactions were initiated by addition of preheated NADPH (1.3 mM) and were allowed to proceed for 30 min. Aliquots (100 µL) were sampled from the incubation mixture (500 µL total volume) at 0 and 30 min. An equal volume of a mixture of MeOH/MeCN (50:50, *v*/*v*, cooled to −20 °C) was added to terminate the reaction. After subsequent homogenization on a vortex mixer (1 min, 21 °C) and centrifugation (10 min, 20,000× *g* rcf, 21 °C), the supernatants (50 µL aliquots) were analyzed via HPLC-UV/VIS (Knauer smartline system equipped with a Rheodyne type 7125 sample injector and a 500 µL sample loop). Chromatographic conditions were as follows. Column: 4.6 mm × 250 mm Kromasil 100-5-C18 (AkzoNobel, Bohus, Sweden); eluent composition: MeCN/H_2_O/AcOH 48:52:0.2 (*v*/*v*/*v*); flow rate: 1 mL/min; detection wavelength: 275 nm. Microsomal assays were performed in quadruplicate.

#### 4.3.2. Metabolite Analysis

Microsomal assays aimed at metabolite profiling were conducted according to the protocol described in Section 4.3.1, but with 10 µL substrate (CBX, MCBX, CPFPX) in a total incubation volume of 1 mL. In Table 6, microsomal protein concentrations and incubation times used in the individual assays are listed. Blank samples containing all matrix components but no substrate were included. Incubations were terminated by adding two volumens of a mixture of MeOH/MeCN (50:50, *v*/*v*, cooled to −20 °C). Samples were then vortexed (1 min, 21 °C), centrifuged (10 min, 20,000× *g* rcf, 21 °C), and evaporated to dryness using a centrifugal vacuum concentrator (Concentrator 5301, Eppendorf, Wesseling-Berzdorf, Germany) set to a temperature of 45 °C. Dried samples were reconstituted with 160 µL HPLC eluent (MeCN/H_2_O/AcOH 35:65:0.1 (*v*/*v*/*v*)) and centrifuged (3 min, 20,000× *g* rcf, 21 °C). Aliquots of the clear supernatant (25 µL) were subsequently injected into the HPLC system. Chromatographic parameters were the same as described in Section 4.3.1, except for the addition of a 3 mm NH_2_ guard column (OPTI-GUARD, Optimize Technologies Inc., Oregon City, OR, USA). For LCMS analyses, the UV-detector outlet was coupled to a mass spectrometer (MSQ PlusTM, Thermo Electron Corporation, San Jose, CA, USA) via an electrospray interface. LCMS parameters were as follows. Nebulizer M gas pressure: 6 bar; desolvation temperature: 500 °C; positive ion mode (ESI+); sprayer voltage: 3000 V; cone voltages: 50 V (unfragmented spectra) or 185 V (fragmented spectra), *m*/*z* range 1–800; scan time: 1 s. Mass spectra were analyzed using Xcalibur software (version 3.0).

#### 4.3.3. Enone Metabolite Formation

In preliminary experiments, the potential enone precursors **5**–**8** (8 µM) were incubated with either RLM (0.4 mg/mL) or HLM (0.8 mg/mL) for up to 4 h according to the protocol given in Section 4.3.1. Multiple samples were taken during incubation and analyzed with regard to the presence of the enone metabolite **4** in the incubation mixture.

The time course of the formation of **4** from precursor **6** was monitored by incubation of **6** (4 µM) with either 1.0 mg/mL HLM for 150 min or 0.4 mg/mL RLM for 100 min according to the procedures described in Section 4.3.1. but with a prolonged centrifugation cycle (15 min) for protein precipitation. Chromatographic separation was performed on a Kromasil C18 column (see Section 4.3.1) equipped with a 3 mm NH_2_ guard column (OPTI-GUARD, Optimize Technologies Inc., Oregon City, OR, USA) using an eluent composed of MeCN/H_2_O/AcOH 45:55:0.1 (*v*/*v*/*v*). All other chromatographic parameters resembled those given above. Incubations were conducted in triplicate.

### 4.4. Data Analysis

Elimination rate constants (*k*) of CBX, MCBX, and CPFPX were derived via linear regression analysis of semi-logarithmic peak area vs. time plots.

In vitro half-life (*t*_1/2_) was calculated as:

(1) $$t_{1/2}=\frac{\ln2}{k}$$

In vitro intrinsic clearance was calculated as follows [42]:

(2) $$Cl_{int}=\frac{\ln2}{t_{1/2}\times c}$$

where *c* is the microsomal protein concentration. Since the main focus of the study was to compare *CL_int_* between species, but not between compounds, no correction for the microsomal unbound fraction was applied.

## 5. Conclusions

Human microsomal metabolism of the three A_1_AR ligands could not be accurately modeled by microsomes of a single animal species. In particular, the closely related rhesus macaque, which represents a popular animal model in pharmacology, exhibited large differences in terms of metabolic activity toward the test compounds. This in turn casts doubts on the usefulness of this species for the pharmacokinetic evaluation and dosimetry of xanthine-derived A_1_AR ligands. By contrast, the beagle dog appears to be a promising preclinical species, especially with regard to in vivo metabolite profiling. The discrepancy between in vitro and in vivo biotransformation of CPFPX in rodents was attributable to the incapacity of the rodent microsomal enzymes to catalyze the final oxidation step leading to the enone metabolite. In conclusion, differences in pharmacokinetics and metabolism of radiolabeled compounds in distinct species should be carefully determined during preclinical development in order to obtain reliable data that can be extrapolated to humans. This is especially important in the context of preclinical dosimetry studies preceding first-in-human clinical trials with new diagnostic or therapeutic radiopharmaceuticals.

## Figures and Tables

**Table 1:** Structural formulae of the ligands and metabolites used in this study. All compounds were synthesized and characterized in-house.

Numbering	Structural Formula	Name, Molecular Weight (MW)
**1**	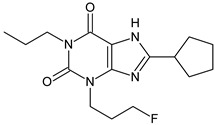	8-Cyclopentyl-3-(3-fluoropropyl)-1-propylxanthine (CPFPX) MW: 322.38 g/mol
**2**	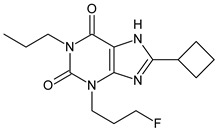	8-Cyclobutyl-3-(3-fluoropropyl)-1-propylxanthine (CBX) MW: 308.35 g/mol
**3**	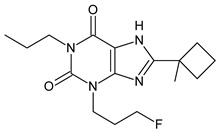	3-(3-Fluoropropyl)-8-(1-methylcyclobutyl)-1-propylxanthine (MCBX) MW: 322.38 g/mol
**4**	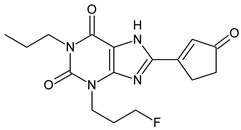	3-(3-Fluoropropyl)-8-(3-oxocyclopent-1-en-1-yl)-1-propylxanthine (CPFPX metabolite, enone metabolite) MW: 334.35 g/mol
**5**	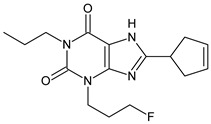	8-(Cyclopent-3-en-1-yl)-3-(3-fluoropropyl)-1-propylxanthine (CPFPX metabolite) MW: 320.36 g/mol
**6**	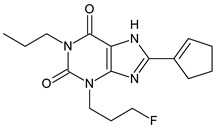	8-(Cyclopent-1-en-1-yl)-3-(3-fluoropropyl)-1-propylxanthine (CPFPX metabolite) MW: 320.36 g/mol
**7**	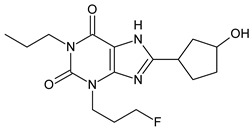	3-(3-Fluoropropyl)-8-(3-hydroxycyclopentyl)-1-propylxanthine (CPFPX metabolite) MW: 338.38 g/mol
**8**	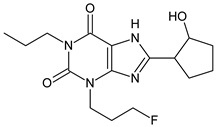	3-(3-Fluoropropyl)-8-(2-hydroxycyclopentyl)-1-propylxanthine (CPFPX metabolite) MW: 338.38 g/mol

**Table 2:** Metabolites of CBX generated in liver microsomes from various species.

Peak	Retention Time (min)	Retention Factor	Functionalization	Site of Functionalization	Interpretation of Fragments ^1^	Species
A1	4.8	0.9	“–OH”	R	b, c, e, f: + OH − H	h, r, m, d, mp, rh
A2	5.3	1.1	n.s.	n.s.	n.s.	(h), r, (m), mp, rh
A3 ^2^	5.6	1.2	“=”: “–OH” ^3^ human 1: 0.5 mini pig 0.05: 1 mouse 0.7: 1 rhesus 0: 1 rat 0.4: 1 dog 0.4: 1	“=” @ R “–OH” @ F(Pr)	“=” d, e: − 2H e,f	h, r, m, d, mp, rh
A4	6.0	1.4	n.s.	n.s.	n.s.	(r), (m), mp, (rh)
A5	9.6	2.9	“–OH”	R	c, e, f: + OH − H	h, r, m, d, mp, rh
A6	10.4	3.2	n.s.	n.s.	n.s.	(h), (r), d, (mp)
A7	12.1	3.9	n.s	n.s.	n.s.	(h), r, m, d, (mp), (rh)
CBX	16.6	5.7	n.a.	n.a	n.a.	(h), r, m, d, (mp), rh

h, human; r, rat; m, mouse; d, dog; mp, mini pig; rh, rhesus; n.s., not specifiable; n.a., not applicable. Brackets indicate minor peaks. ^1^ according to Figure 5. ^2^ coelution of two metabolites. ^3^ ratio of [M + H]^+^ intensities (*m*/*z* 307 & 325), determined from unfragmented spectra.

**Table 3:** Metabolites of MCBX generated in liver microsomes from various species.

Peak	Retention Time (min)	Retention Factor	Functionalization	Site of Functionalization	Interpretation of Fragments ^1^	Species
B1	5.3	1.1	n.s.	n.s.	n.s.	r, mp
B2	5.7	1.3	n.s.	n.s.	n.s.	(r), m, (d), (mp), (rh)
B3	6.3	1.5	“–OH”	R	d: + OH − 2 H	h, r, m, d, mp, rh
B4	7.1	1.8	n.s.	n.s.	n.s.	(h), m, d, (rh)
B5	8.0	2.2	“–OH”	R	b, d: + OH − 2 H	h, r, m, d, mp, rh
B6	8.6	2.4	“–OH”	F(Pr)	d, e	h, r, m, d, mp, rh
B7	8.9	2.6	“–OH”	R	c, d: + OH − 2H	h, r, m, d, mp, rh
B8	12.9	4.2	n.s.	n.s.	n.s.	(h), m, d, mp, (rh)
B9	21.7	7.7	n.s	n.s.	n.s.	h, r, m, d, (mp), (rh)
B10	31.6	11.7	n.s	n.s.	n.s.	h, r, m, d, (mp), (rh)
MCBX	33.1	12.3	n.a.	n.a.	n.a.	h, r, m, d, mp, rh

h, human; r, rat; m, mouse; d, dog; mp, mini pig; rh, rhesus; n.s., not specifiable; n.a., not applicable. Brackets indicate minor peaks. ^1^ according to Figure 5.

**Table 4:** Metabolites of CPFPX generated in liver microsomes from various species.

Peak	Retention Time (min)	Retention Factor	Functionalization	Site of Functionalization	Interpretation of Fragments ^1^	Species
C1	5.5	1.2	“–OH”	R	b, e, f: + OH − H	h, r, m, d, mp, rh
C2	5.9	1.4	“–OH”	R	f: + OH − H	h, r, m, d, mp, rh
C3	6.3	1.5	“=O”	R	b, e, f: + O − H	h, r, m, d, (mp), (rh)
C4	7.4	2.0	n.s.	n.s.	n.s.	(h), r, m, d, mp, rh
C5	7.9	2.2	“=”	(F)Pr	d, e, f	h, r, m, d, mp, rh
C6	8.3	2.3	“=”	(F)Pr	e, f	h, r, m, d, mp, rh
C7	8.7	2.5	n.s.	n.s.	n.s.	(h), r, (m), (d), (rh)
C8	9.0	2.6	n.s.	n.s.	n.s.	(r), m, (d), (rh)
C9	9.8	2.9	“=O”/“=”	R	b, e, f: + OH − 4H	h, d
C10	12.2	3.9	n.s	n.s.	n.s.	h, r, m, d, mp, rh
C11	21.4	7.6	n.s.	n.s.	n.s.	(h), r, m, (mp), rh
C12	34.0	12.6	n.s	n.s.	n.s.	r, (m), (d)
CPFPX	29.4	10.8	n.a.	n.a.	n.a.	h, r, m, d, mp, rh

h, human; r, rat; m, mouse; d, dog; mp, mini pig; rh, rhesus; n.s., not specifiable; n.a., not applicable. Brackets indicate minor peaks. ^1^ according to Figure 5.

**Table 5:** Total microsomal P450 content reported for various species.

Species	Total Microsomal P450 Content (nmol/mg Microsomal Protein)				
	[13]	[14]	[15]	[16]	[17]
Human	0.307 ± 0.160	0.231 ± 0.013	0.31 ± 0.09	n.d.	0.29 ± 0.06
Rat	0.673 ± 0.050	0.444 ± 0.016	0.58 ± 0.02	n.d.	n.d.
Mouse	n.d.	0.719 ± 0.041	0.48 ± 0.04	n.d.	n.d.
Mini pig	n.d.	n.d.	n.d.	0.798 ± 0.145	n.d.
Dog	0.385 ± 0.036	0.685 ± 0.031	n.d.	n.d.	n.d.
Monkey	1.030 ± 0.106 ^1^	1.195 ± 0.089 ^2^	0.74 ± 0.02 ^1^	n.d.	0.95 ± 0.08 ^2^

n.d., not determined; ^1^ cynomolgus monkey; ^2^ rhesus monkey.

**Table 6:** Incubation conditions for generation of in vitro metabolite profiles.

Microsomes	Substrate	Microsomal Protein Concentration (mg/mL)	Incubation Time (min)
HLM	CBX, MCBX, CPFPX	0.8	180
RLM	CBX, MCBX, CPFPX	0.4	30
MLM	CBX, MCBX, CPFPX	0.4	30
DLM	CBX, MCBX, CPFPX	0.8	45
MPLM	CBX	0.8	45
	MCBX, CPFPX	0.8	30
RMLM	CBX	0.04	45
	MCBX, CPFPX	0.04	30

## Data Availability

The data presented in this study are available on request from the corresponding author.
